# The mental burden of stay-at-home order extensions during COVID-19

**DOI:** 10.1038/s41598-024-54059-z

**Published:** 2024-02-21

**Authors:** Michelle S. Segovia, Samir Huseynov, Marco A. Palma, Rodolfo M. Nayga

**Affiliations:** 1 Department of Applied Economics and Statistics, University of Delaware, 204 Townsend Hall, Newark, DE 19716 USA; 2 Department of Agricultural Economics and Rural Sociology, Auburn University, 202 Comer Hall, Auburn, AL 36849 USA; 3 Department of Agricultural Economics, Texas A&M University, 2124 TAMU, College Station, TX 77843 USA; 4 Korea University, Seoul, South Korea

**Keywords:** Human behaviour, Public health

## Abstract

This study evaluates the psychological impact of stay-at-home extension orders during COVID-19 and its relationship with individuals’ expectations on the duration of the extensions. An online survey was administered to 1259 US adult residents to measure symptoms of post-traumatic stress disorder (PTSD), anxiety and stress induced by different stay-at-home order extensions using hypothetical length scenarios. We find that individuals exposed to two 2-week order extensions exhibit higher levels of stress and anxiety compared to those exposed to a single 4-week extension. We also find that subjects with longer expected extensions exhibit more signs of psychological damage than those with shorter expected extensions. Furthermore, we find that the negative psychological consequences of providing two shorter extensions is observed only among subjects with extension expectations of four weeks or less. Our results demonstrate that people’s expectations affect the level of psychological damage caused by lockdown mandates. Our findings suggest that whenever lockdown extensions are necessary, reduced psychological distress may be possible by implementing a one-time restriction, rather than extending multiple smaller extensions perhaps due to manipulation of personal expectations.

## Introduction

The rapid and widespread surge in COVID-19 prompted government officials worldwide to mandate stay-at-home lockdown restrictions (i.e., stay-at-home orders, curfews, quarantines) to reduce the spread of the virus. In the United States (US), the first confirmed coronavirus case was announced on January 21 of 2020, and by mid-March, government authorities in more than 20 US states had issued stay-at-home orders directing all residents to self-quarantine or isolate at home. This mass quarantine strategy was designed to ameliorate the devastating effects of the pandemic and slow down the rate of hospitalizations and prevent a collapse of the health care system^1^. [Quarantine differs from isolation, which is the separation of people who have been diagnosed with a contagious disease from people who are not sick; however, they are often used interchangeable^2^. In this paper, we use both terms interchangeably.]

Although quarantine or isolation may be a necessary preventive measure, it often has considerable psychological, emotional, and financial impacts^3–5^. For example^6^, suggest the presence of negative psychological consequences, including post-traumatic stress disorder (PTSD) symptoms, confusion, anger, and mental health damage directly associated with quarantining. It was anticipated by scientists that the outbreak of COVID-19 and consequent preventive lockdown restrictions would cause mental health problems^7–11^. In fact, recent studies provide preliminary evidence of significantly elevated levels of mental distress, depression, anxiety, and post-traumatic stress among both the general public and medical personnel due to COVID-19^12–24^. In the US, more than one-third of Americans (36%) report that coronavirus has a serious impact on their mental health, according to a national poll by the American Psychiatric Association^25^. [Polls by the Kaiser Family Foundation and a National Public Radio found similar results^10,26^.] Likewise^8^, suggests that the US population is worried, fearful, and uncertain about the pandemic and the consequences it will have for themselves, their families, and communities.

One of the main stressors during quarantine measures is the length of time for the isolation or lockdown period^6,27,28^. Longer quarantine durations are associated with larger adverse psychological consequences, in particular post-traumatic stress symptoms, avoidance behaviors and anger^27,28^. In this regard,^29^ provides anecdotal evidence that the negative psychological consequences are magnified when increasingly long projections for isolation are given by government officials and health experts; that is, short periods of isolation might be far easier for people to accept and conform to than indefinite timelines without a clear endpoint. The author relates this effect to constant changes in people’s expectations about the ending date of the lockdown, where the *uncertainty* in itself may increase the level of mental and emotional damage. Although the association between individuals’ expectations of the duration of the quarantine directive and their stress and mental wellbeing has been previously established in the context of military deployment^30^, the nature of this relationship for the public during outbreaks like COVID-19 is yet to be assessed. To our knowledge, this study provides the first attempts at examining the mental health effects of stay-at-home extension mandates and its relationship with individual’s expectations on extensions duration.

We contribute to the related literature by investigating the effect of inducing different stay-at-home extension lengths on the mental health of individuals during the COVID-19 pandemic using exogenously assigned hypothetical scenarios. [On average, US residents had been under a stay-at-home order for a period of 29 days when the study was initiated (April 30, 2020). These initial stay-at-home orders have been extended by state officials in over 25 US states^31^]. More specifically, we conduct an online survey where participants are exogenously induced with a hypothetical scenario in which a 4-week stay-at-home order has taken place in the state they reside. Subjects are randomly assigned to one of three hypothetical scenario conditions: (1) *One extension* treatment, in which subjects are required to stay-at-home for an additional four weeks; (2) *Two extensions* treatment, in which two stay-at home extensions are implemented, each one for two weeks; and (3) *No extension* or *baseline*, where no order extension is announced. We examine the effect of these hypothetical stay-at-home order extensions on three mental health outcomes: individuals’ stress or distress level, PTSD symptoms, and anxiety. We randomly assigned subjects to these hypothetical scenarios in an attempt to estimate their causal effects relative to the baseline of no extension. Similar hypothetical scenarios to study mental function have been previously implemented and the methodology has been effective in inducing an altered mental state^32,33^. Finding signs of PTSD, stress and anxiety effects on this short-term hypothetical scenario interventions provides a lower bound activation of the likely effects of actual stay-at-home extensions. Although the main objective of the extensions is to block the spread of the virus, the results are useful for designing protocols that consider mental health as one of the relevant outcomes. We also examine these effects in relation to subjects’ prior extension expectations. To obtain individual level prior expectations on stay-at-home order extensions, we ask participants to report the number of weeks (if any) they would expect the 4-week stay-at-home order to be extended by their state government.Prior expectations of stay-at-home order extensions are measured after subjects are exposed to the 4-week hypothetical stay-at-home order, and before receiving the manipulation. Our findings show that for the equivalent 4-week stay-at-home mandate, subjects in the *Two extensions* treatment exhibit higher levels of distress and anxiety compared to those in the *One extension* treatment and the *baseline*. No significant effects are found for the *One extension* treatment compared to the *baseline.* We also find that people who expect a stay-at-home extension order of more than four weeks exhibit higher levels of distress, PTSD symptoms, and anxiety compared to those with expectations of less than four weeks.

The results from our study highlight the potential mental health effects of stay-at-home order extensions for equivalent length-periods, particularly those provided as multiple smaller extensions. If order extensions become strictly necessary during pandemics (and perhaps during subsequent waves of pandemics), our results suggest that consecutive stay-at-home order extensions might be more psychologically detrimental than imposing just a one-time lockdown restriction, with similar total duration in both cases. It is also likely that if longer mandates are imposed that are not necessary, terminating them early may have some positive effects on mental health. While this is an area that still needs more research, our results are important for designing future directives for pandemic lockdowns. Enhancing the mental well-being of individuals during periods of isolation is crucial in the continuing fight against the disease.

### Mental health outcomes of quarantine

Quarantine and isolation are public health measures used to contain and control the spread of infectious diseases. While isolation separates infected individuals from those who are not sick, quarantine separates and restricts the movement of people who are presumed to have been exposed to a contagious disease to ascertain if they become unwell^34^. However, both terms are often used interchangeably particularly when communicating with the public. Historically, quarantine was used to prevent the transmission of infectious diseases during the fourteenth century such as the plague in Europe^35^. [The term *quarantine* derives from the Italian words *quaranta*, which means 40 days and referred to a 40-day period of isolation for certain ships entering the Venice port during the fourteenth and fifteenth centuries^36^.] Since then, public health practices became increasingly used to combat infectious epidemics globally, including cholera, yellow fever, and more recently, the Severe Acute Respiratory Syndrome (SARS) and COVID-19 outbreaks. [Refer to^1^ for a summary on the history of quarantine.]

Regardless of the success of quarantine and isolation in particular circumstances, these measures may create heavy psychological, emotional, and financial hardships for people experiencing such restrictions^37–40^. Prevalent mental health problems among individuals isolated due to risk of infection include depression, anxiety, mood disorders, psychological distress, PTSD, insomnia, fear, among other adverse psychological effects^41^. For example^42^, examined the mental health status of 1656 people quarantining due to the Middle East Respiratory Syndrome (MERS) epidemic and found that 7.6% and 16.6% of them exhibited symptoms of anxiety and anger, respectively. The prevalence of these symptoms, although at lower rates, was still prevalent at four to six months after release from isolation. A review by^6^ includes studies examining the mental health and psychological wellbeing of quarantined people during infectious diseases such as SARS, Ebola, H1N1 influenza, MERS, and Equine influenza. Patients reported symptoms of depression, stress, anger, guilt, sadness, and irritability. Isolated children presented PTSD scores four times higher than those not quarantined, while 28% of parents quarantined had trauma related mental disorders compared to 6% of parents who were not quarantined. Likewise, adverse mental health conditions among healthcare providers who worked under quarantine include acute stress disorder, anxiety, depression, irritability, insomnia, detachment, and PTSD symptoms, even 3 years after the quarantine period. The negative mental health outcome on healthcare workers has also been reported during the COVID-19 pandemic^37^. Findings from similar studies show that quarantined and isolated individuals often meet the criteria of post-traumatic stress disorder. For example, a web-based survey evaluated the mental health of individuals quarantining during the 2003 SARS outbreak in Canada, finding symptoms of post-traumatic stress disorder and anxiety in 28.9% and 31.2% of respondents, respectively. Importantly, an increased prevalence of PTSD and depressive symptoms was observed for longer durations of quarantine and subjects with acquaintance or exposure to an infected person^28^. This finding goes in line with that by^27^ suggesting a positive correlation between high prevalence of PTSD symptoms and longer quarantine periods, increased compliance with directives, and healthcare worker status during the SARS outbreak.

Most recently, mass quarantine has been used to control the spread of COVID-19 especially during the earlier stages of the pandemic. As a result, researchers have become interested in the mental health outcomes of populations affected by the pandemic and related quarantine. For example^5^, evaluated the prevalence of psychological distress within infected patients, individuals under quarantine and the public. An increased prevalence of depression was found among infected patients, while there was no significant difference in the anxiety level across the three groups. Both infected patients and the public were more likely to demonstrate depressed mood, somatic symptoms and anxiety-like behavior compared to individuals under quarantine. Paralleling with findings by^15,19,23^, the study by^8^ showed high levels of worry and fear toward COVID-19 among the US population, especially in regions with the highest number of confirmed cases. These studies also reported bivariate relationships between socially vulnerable respondents (i.e., female, Hispanic, Asians, foreign born individuals) and fear, depression, and anxiety. [The mental health impacts of pandemic-driven social and psychical isolation on vulnerable populations, including adolescents, elderly people, homeless people, and people with disabilities have also been addressed by previous studies^12,43–45^.]

In summary, results from previous research indicate that quarantine have negative impacts on individuals’ mental health. These effects are being reported during the current COVID-19 pandemic. Relatively few studies have examined the psychological impacts of quarantine duration and extensions, but evidence suggests that longer durations of quarantine and repeated lockdown extensions are likely to exacerbate negative mental health effects^46–49^. Our study not only examines the psychological impact of stay-at-home extension orders during COVID-19, but also investigates its relationship with individuals’ expectations on the duration of the extensions. [The role of individuals’ expectations on lockdown length was exploratory in nature. We expect to see a larger negative impact on mental wellbeing as the gap between the expectation of the lockdown duration and the actual duration increases. We expect to observe this effect based on the large economic evidence showing that an expectation-realization gap impacts the influence that information has on decision-making^50,51^. The channel for the additional mental toll may be that people form expectations for a particular lockdown length and when it is extended, it overpasses the expectations of individuals creating a larger mental health effect.]

## Methods

### Experimental design

The online experiment followed a between-subjects design where the number of stay-at-home order extensions was the manipulating factor. Participants randomly received one of three stay-at-home order extension scenarios: (1) *No extension* or *baseline* (n = 364), (2) *One extension* (n = 360), and (3) *Two extensions* (n = 535). In all treatment scenarios, respondents were asked to imagine that a 4-week stay-at-home order has taken place in the state they reside. For the *One extension* treatment, respondents were further informed that the stay-at-home order has been re-evaluated before its expiration date and a 4-week extension has been issued, for a total of an 8-week stay-at-home order. For the *Two extensions* treatment, respondents were instead informed that the order has been re-evaluated and two separate extensions, each lasting 2 weeks, have been issued. Specifically, following the initial 4-week stay at home order, a 2-week extension was announced for a total of 6-week order. Subsequently, another 2-week extension was issued for an 8-week stay-at-home order total.[Subjects had no knowledge of the second extension at the moment the first extension was announced.] For the *No extension* condition, the 4-week stay-at-home was announced without any further extension. Notice that the total stay-at-home order duration time was the same in the two treatments—i.e., 8 weeks; this makes the number of extensions the only changing factor (between treatments) in the experimental design. [The order duration for the *baseline* was selected to mimic the way in which initial stay-at-home orders were issued by US state officials during the first phase of the pandemic. For example, Alaska was the first state to allow the order to expire on April 24, 2020, after 4 weeks of becoming effective (March 28, 2020); similar lockdown durations were observed for the states of Alabama, Georgia, Florida, Idaho, Maine, Missouri, Pennsylvania, Montana, Utah, among others^31,52^. We acknowledge that our baseline varied in both the lockdown duration and the presence of extensions compared to the treatments; the implications of these differences are discussed in the results.] As the stay-at-home order duration varied by state at the time the survey was implemented, the scenarios used in the study are hypothetical. We followed the literature and implemented a COVID-19 related cheap talk prior to the manipulation in an attempt to mitigate hypothetical bias in our results^53,54^. The cheap talk consisted of a statement communicating subjects about situations in which people might respond to mandates in a hypothetical scenario differently than in a real situation and inducing them to behave in the same way that they would if the decision to adhere to COVID-19 regulations was real. Moreover, we constantly reminded subjects about the importance of providing truthful responses throughout the experiment. Finding signs of PTSD, stress and anxiety on these short-term hypothetical scenarios provides a lower bound activation of the likely effects of actual stay-at-home extensions. The scenarios provided to participants were realistic because many US states (and countries) at the time faced uncertainty about how to establish and the duration of stay-at-home orders.

The scripts for the three scenarios and cheap talk are displayed in S1 Appendix.

### Procedures

We implemented a web-based survey from April 30th to May 20th of 2020.[A pilot test was sent to 51 respondents to ensure the instrument was working appropriately.] Respondents were US adult residents drawn from the pool of Dynata’s database registered participants. A total of 1259 participants completed the experiment.[Dynata offers incentives to panelists through their preferred currency or points that can be redeemed for cash, gift cards, charity donations, airline miles and prize draw entries.] During the survey period, most of the US states had mandated stay-at-home orders as the number of confirmed cases rose above 1.3 million.[Information on state-at-home orders by state was retrieved from^31^.] On average, participants had been under some sort of lockdown regulation for about 37 days (s.d. = 8.9 days) at the time of the survey and there was uncertainty about the possible duration of the mandates which is crucial in the implementation of our induced hypothetical scenarios. [The high degree of uncertainty experienced during the early stages of the pandemic was evident in the large variation on the public’s perception and acceptance of lockdown restrictions. A poll conducted by the Kaiser Family Foundation in April of 2020 reported that 80% of Americans agreed with the implementation of strict stay-at-home guidelines to control the spread of the virus; however, willingness to adhere to mandates varied significantly, from 37% of respondents saying they could obey the restrictions for another 1–3 months, 34% for more than 6 months, while only 3% reported they could not adhere to regulations at all^55^.] The study received ethical approval from the University of Missouri Institutional Review Board (IRB). The survey was implemented in accordance with relevant guidelines and regulations, and informed consent was obtained from all participants.

**Figure 1:**
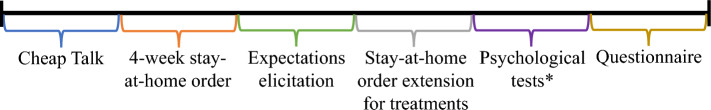
Experimental procedure. *The psychological tests were measured after each 2-week extension in the *Two extensions* treatment, i.e., at 6-week stay-at-home order and 8-week order.

At the beginning of the experiment, subjects were asked to imagine that a 4-week stay-at-home order has been placed in the state they reside (see Fig. 1 for the experiment timeline). To assess subjects’ expectations on stay-at-home order extensions, we asked them to report the number of weeks (if any) they would expect this 4-week stay-at-home order to be extended by the state government. This was done prior to subjects receiving the manipulation (i.e., extension scenarios) in order to align their prior expectations with the actual treatment extensions. Subjects then received their assigned treatment followed by questions regarding psychological measures on the impact of stay-at-home order extensions. The psychological impact of stay-at-home order extensions was evaluated using three validated psychological instruments: the Perceived Stress Scale (PSS-10;^56^), the Impact of Events Score-Revised (IES-R;^57^), and the Generalized Anxiety Disorder-7 (GAD-7;^58^). In the *No extension* baseline and *One extension* treatment, subjects completed the three psychological tests one time, following the 4-week stay-at-home order and 8-week order respectively. In the *Two extension* treatment, subjects completed the three tests twice, after each 2-week extension (i.e., at 6-week order and 8-week order). First, subjects answered the PSS-10 instrument, which is a 10-item self-report measure of global perceived stress designed to determine how unpredictable, uncontrollable, and overloaded individuals find their lives^56^. Subjects were asked to rate the occurrence of each statement happening to them as a consequence of the lockdown extension on a 5-point scale (0 = Never; 4 = Very often). A total score ranging from 0 to 40 was calculated by reverse scoring the four positively worded items (Items 4, 5, 7, and 8) and then summing all the scale items. Higher scores are indicative of greater perceived distress/stress feelings^59^.[Subscale scores can also be computed by summing the six negative worded items (Items 1, 2, 3, 6, 9, and 10) and the four positive worded items (Items 4, 5, 7, and 8) separately. A description of this approach can be found in^59^.] Symptoms of post-traumatic stress disorder (PTSD) were then elicited using the self-reported IES-R^27,28,57^, which allowed us to assess the subjective distress resulting from the COVID-19 quarantine experience *after* the additional stay-at-home order extension. Responses to 22 items, each with a rating scale from 0 to 4 (0 = Not at all; 4 = Extremely), were scored and summed to a score of 88. A score of ≥ 20 on the IES-R was used to estimate the prevalence of PTSD symptoms^28,60^.[Previous studies have also separated the IES-R instrument into three subscales: the avoidance scale, intrusion scale, and the hyperarousal scale^57^.] Furthermore, respondents’ anxiety level due to the state-at-home order extension was measured using the Generalized Anxiety Disorder-7 (GAD-7)^58,61^. They were asked to rate the occurrence of each anxiety symptom in response to the stay-at-home order extension on a 4-point scale (0 = Not at all; 3 = Nearly every day). Total scores range from 0 to 21, with higher scores indicating greater severity anxiety. According to^58^, the total score may be categorized into four severity groups: minimal (0–4), mild (5–9), moderate (10–14) and serious (14–20).Following^62^, subjects were also asked to rate their anticipated worry (a prospective measure), experienced worry (a retrospective measure), current worry (a current measure), perceived absolute susceptibility (a prospective measure), and perceived relative susceptibility (a prospective measure) about the coronavirus. Finally, subjects were asked information regarding their actual COVID-19 quarantine experience and their socio-economic characteristics. All experimental instructions are available in S2 Appendix.

## Results

We received responses from 1259 adults from 49 US states. Approximately, 40% of the subjects are male, with an average age of 45 years and average annual household income of $77,718 (median income of $75,000). Two-thirds of participants identified themselves as Caucasian, 15% as African American, 8% as Hispanic, and 10% as “other”. About 17% of the participants indicated that they had lost their jobs and 42% had incurred in financial losses due to COVID-19. Moreover, 21% (36%) indicated living with an elder (children) in their household. About 33% of the subjects reported being affiliated to the Republican Party, 44% to the Democratic Party, and 23% to other or no political affiliation. Table 1 displays the demographic profile of participants along with a balance check across treatments. There are no statistical differences in the proportion of Hispanics, rural residents, and individuals living with an elder in their household across treatments (*p* > 0.10 for all Kruskal–Wallis rank tests). Moreover, no differences are found in household size and individuals’ risk attitudes across treatments (p > 0.10 for all Kruskal–Wallis rank tests).

**Table 1:** Balance check across treatment groups.

Variable	Description	Mean (std. err.)	Mean (std. err.)	Mean (std. err.)	Kruskal–Wallis test
		No extension	One extension	Two extensions	
Age	Age in years, 18–99	46.60 (0.82)	47.80 (0.87)	42.80 (0.63)	p < 0.01
Male	DV = 1 if male, 0 otherwise	0.47 (0.03)	0.44 (0.03)	0.32 (0.02)	p < 0.01
Income	Yearly income	79,821.00 (2356.00)	76,528.00 (2417.00)	101,813.00 (2146.00)	p < 0.01
Household size	Number of household members	2.65 (0.14)	2.63 (0.07)	2.63 (0.06)	p = 0.30
White	DV = 1 if white, 0 otherwise	0.65 (0.03)	0.70 (0.02)	0.44 (0.02)	p < 0.01
African American	DV = 1 if African American, 0 otherwise	0.16 (0.02)	0.14 (0.02)	0.10 (0.01)	p = 0.03
Hispanic	DV = 1 if Hispanic, 0 otherwise	0.08 (0.01)	0.07 (0.01)	0.06 (0.01)	p = 0.35
Other race	DV = 1 if Native American, Asian or 'other' race, 0 otherwise	0.10 (0.02)	0.09 (0.02)	0.06 (0.01)	p = 0.04
Republican party	DV = 1 if Republican party affiliated, 0 otherwise	0.28 (0.02)	0.34 (0.03)	0.24 (0.02)	p < 0.01
Democratic party	DV = 1 if Democratic party affiliated, 0 otherwise	0.50 (0.03)	0.43 (0.03)	0.25 (0.02)	p < 0.01
Other party	DV = 1 if 'none' or 'other' party affiliated, 0 otherwise	0.22 (0.02)	0.24 (0.02)	0.17 (0.02)	p = 0.03
Rural	DV = 1 if rural resident, 0 otherwise	0.15 (0.02)	0.18 (0.02)	0.20 (0.02)	p = 0.15
Living with elder	Living with elder (65 + years old) in household	0.22 (0.02)	0.21 (0.02)	0.20 (0.02)	p = 0.63
Risk seeking	Risk seeking, 0–10	5.46 (0.14)	5.33 (0.13)	5.38 (0.14)	p = 0.78
Lockdown length	Number of days in lockdown	35.64 (0.39)	34.95 (0.41)	40.48 (0.41)	p < 0.01
N		364	360	535	

Participants were also asked to report the state in which they reside during the time of the survey, which was used to calculate the number of days that they had been under lockdown.[Lockdown duration is calculated using information on state-at-home orders by state retrieved from^31^.] On average, respondents had been under lockdown for 36, 35, and 40 days in the *No extension*, *One extension*, and *Two extension* conditions, respectively. Since the duration of lockdown at the time of the survey varies across treatments (*p* < 0.01 for Kruskal–Wallis rank test), we control for this effect in the regression analysis below.[We also find statistical differences in age, gender, income, political affiliation and the proportion of White and African-American individuals across treatments, which we control for in the regression models in Tables 2 and 3.]

**Table 2:** OLS regressions on psychological measures.

	PSS-10 (1)	PSS-10 (2)	PSS-10 (3)	IES-R (4)	IES-R (5)	IES-R (6)	GAD-7 (7)	GAD-7 (8)	GAD-7 (9)
One extension	0.041 (0.534)	0.011 (0.531)	− 0.070 (0.505)	− 0.308 (1.605)	− 0.428 (1.588)	− 0.516 (1.417)	− 0.544 (0.455)	− 0.574 (0.452)	− 0.546 (0.412)
Two extensions	1.666*** (0.488)	1.594*** (0.485)	1.217** (0.506)	0.443 (1.467)	0.150 (1.452)	0.124 (1.422)	0.899** (0.416)	0.824** (0.413)	0.767* (0.414)
Expect more than 4 week extension		1.771*** (0.454)	1.574*** (0.481)		7.205*** (1.358)	6.016*** (1.351)		1.857*** (0.386)	1.520*** (0.393)
Justifiable			− 1.857*** (0.474)			− 2.532* (1.329)			− 0.917** (0.387)
Sufficient			0.539 (0.430)			4.823*** (1.207)			0.626* (0.351)
Rural			− 0.457 (0.566)			− 2.871* (1.589)			− 0.798* (0.462)
Living with elder			0.697 (0.529)			4.254*** (1.484)			1.113** (0.432)
Risk seeking			0.016 (0.083)			1.571*** (0.234)			0.249*** (0.068)
Other polit. affiliation			− 0.820 (0.538)			− 6.659*** (1.510)			− 1.695*** (0.439)
Republican affiliation			− 0.738 (0.503)			0.262 (1.411)			− 0.030 (0.411)
Age			− 0.126*** (0.015)			− 0.356*** (0.041)			− 0.119*** (0.012)
Male			− 1.475*** (0.446)			− 1.315 (1.253)			− 0.797** (0.365)
Income			− 0.000** (0.000)			0.000 (0.000)			0.000 (0.000)
African American			− 1.800*** (0.616)			0.695 (1.729)			− 0.601 (0.503)
Hispanic			0.588 (0.797)			3.431 (2.238)			0.141 (0.651)
Other race			− 0.122 (0.723)			− 1.871 (2.029)			− 0.817 (0.590)
Lockdown length			0.030 (0.027)			− 0.075 (0.074)			− 0.020 (0.022)
Constant	16.184*** (0.376)	15.746*** (0.391)	23.737*** (1.447)	29.808*** (1.132)	28.026*** (1.169)	39.631*** (4.063)	6.332*** (0.321)	5.873*** (0.332)	11.976*** (1.182)
Observations	1259	1259	1076	1259	1259	1076	1259	1259	1076
R^2^	0.013	0.025	0.154	0.002	0.022	0.225	0.010	0.028	0.195
F statistic	8.089***	10.529***	11.298	0.136	9.476***	18.076***	6.317***	11.996***	15.106***
D.F	(2; 1256)	(3; 1255)	(17; 1058)	(2; 1256)	(3; 1255)	(17; 1058)	(2; 1256)	(3; 1255)	(17; 1058)

***p < 0.01, **p < 0.05, and *p < 0.10; scores for the *Two extension* treatment correspond to those elicited after the second 2-week extension.

**Table 3:** OLS regressions on psychological measures by expectation category.

	PSS-10 (4 weeks or less) (1)	PSS-10 (more than 4 weeks ) (2)	IES-R (4 weeks or less) (3)	IES-R (more than 4 weeks ) (4)	GAD-7 (4 weeks or less) (5)	GAD-7 (more than 4 weeks ) (6)
One extension	− 0.101 (0.594)	− 0.253 (1.018)	0.112 (1.623)	− 2.728 (2.998)	− 0.407 (0.480)	− 1.332 (0.845)
Two extensions	1.193** (0.596)	1.076 (0.999)	0.692 (1.630)	− 1.822 (2.942)	0.968** (0.482)	− 0.132 (0.829)
Justifiable	− 1.981*** (0.546)	− 1.229 (1.006)	− 3.272** (1.494)	− 0.534 (2.964)	− 1.217*** (0.442)	0.168 (0.836)
Sufficient	0.555 (0.500)	0.178 (0.885)	3.702*** (1.367)	7.857*** (2.608)	0.265 (0.405)	1.618** (0.735)
Rural	0.129 (0.662)	− 1.716 (1.124)	− 1.904 (1.810)	− 4.451 (3.312)	− 0.401 (0.536)	− 1.550* (0.934)
Living with elder	0.783 (0.639)	0.340 (0.989)	3.277* (1.747)	3.789 (2.913)	1.056** (0.517)	0.680 (0.821)
Risk seeking	− 0.100 (0.100)	0.307* (0.158)	1.194*** (0.272)	2.322*** (0.465)	0.152* (0.081)	0.452*** (0.131)
Other polit. affiliation	− 0.804 (0.628)	− 0.775 (1.120)	− 7.464*** (1.717)	− 2.897 (3.299)	− 1.829*** (0.508)	− 0.755 (0.930)
Republican affiliation	− 0.386 (0.594)	− 1.939** (0.967)	− 0.851 (1.625)	3.262 (2.848)	0.070 (0.481)	− 0.519 (0.803)
Age	− 0.126*** (0.018)	− 0.127*** (0.027)	− 0.305*** (0.049)	− 0.434*** (0.080)	− 0.110*** (0.015)	− 0.133*** (0.023)
Male	− 1.668*** (0.531)	− 1.076 (0.847)	− 1.977 (1.452)	0.146 (2.495)	− 0.964** (0.430)	− 0.497 (0.703)
Income	− 0.000 (0.000)	− 0.000** (0.000)	− 0.000 (0.000)	0.000 (0.000)	0.000 (0.000)	0.000 (0.000)
African American	− 1.870** (0.770)	− 1.615 (1.046)	3.132 (2.105)	− 3.464 (3.081)	− 0.179 (0.623)	− 1.431 (0.869)
Hispanic	0.893 (0.982)	0.193 (1.389)	5.079* (2.684)	1.940 (4.091)	0.794 (0.794)	− 0.855 (1.153)
Other race	− 0.509 (0.842)	0.948 (1.454)	− 1.900 (2.303)	− 0.975 (4.283)	− 0.814 (0.682)	− 0.811 (1.207)
Lockdown length	0.019 (0.032)	0.050 (0.048)	− 0.049 (0.088)	− 0.141 (0.143)	− 0.021 (0.026)	− 0.015 (0.040)
Constant	24.392*** (1.728)	23.933*** (2.735)	40.336*** (4.727)	41.649*** (8.055)	12.281*** (1.399)	12.140*** (2.271)
Observations	798	278	798	278	798	278
R^2^	0.137	0.187	0.155	0.347	0.151	0.279
F Statistic	7.731***	3.758***	8.954***	8.677***	8.684***	6.310***
D.F	(16; 781)	(16; 261)	(16; 781)	(16; 261)	(16; 781)	(16; 261)

***p < 0.01, **p < 0.05, and *p < 0.10; scores for the *Two extension* treatment correspond to those elicited after the second 2-week extension.

We start by discussing our main results on the impact of the number of stay-at-home order extensions on the mental wellbeing of participants. For the *Two extension* treatment, we present the results from the psychological measures elicited after the second 2-week extension (i.e., 8-weeks order duration) in the main text, and relegate the results for the first 2-week extension (i.e., 6-weeks order) into S3 Appendix. We relegate the first extension results because most of the treatment effects replicate what we find after the second extension was announced. We also do so to better align our analysis of treatment effects and individuals’ prior expectations of lockdown duration (8 weeks total).

**Result 1.** *Providing two 2-week extensions increases individuals’ distress and anxiety compared to one 4-week extension and the no extension baseline.*

**Figure 2:**
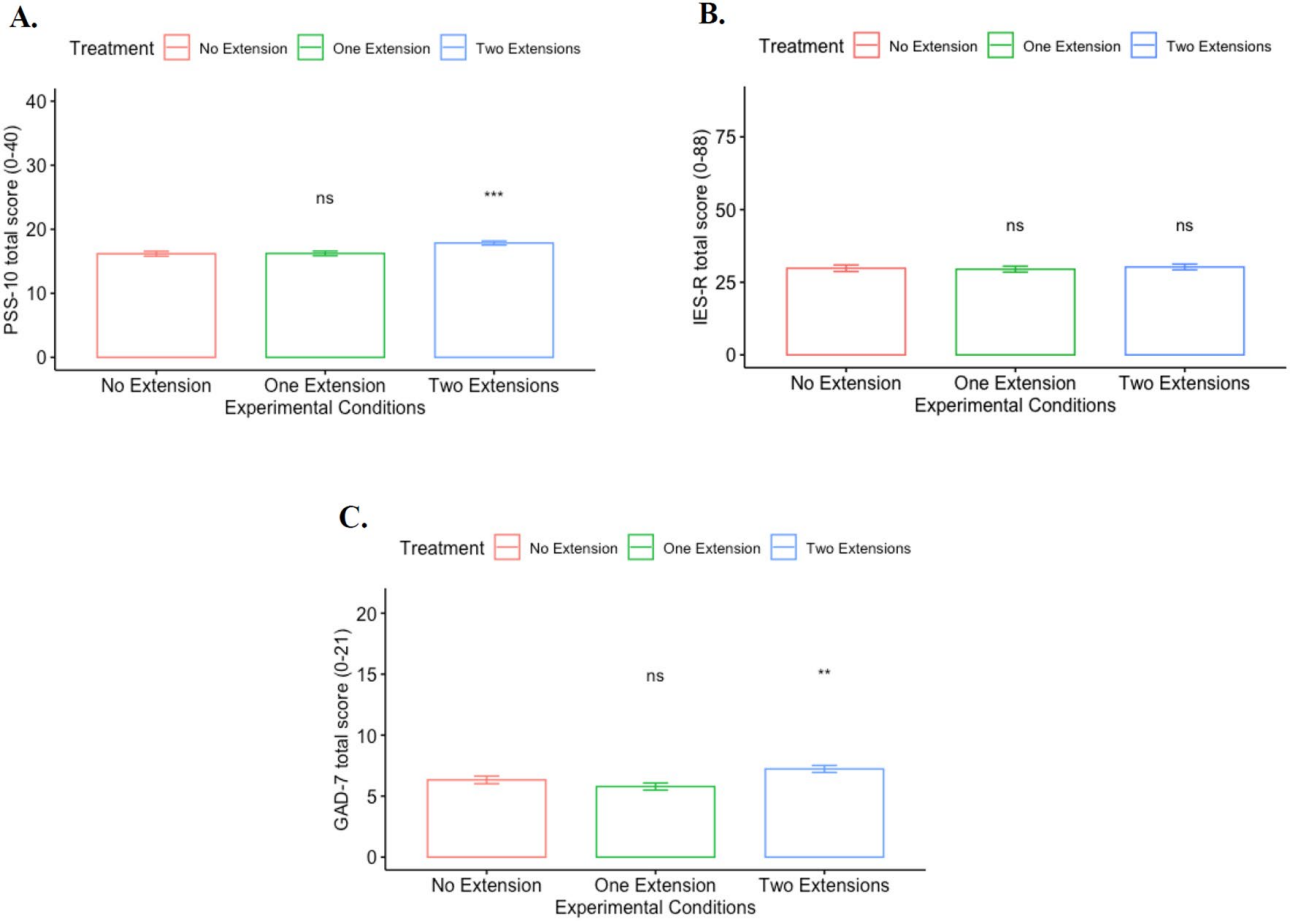
Psychological total scores by treatment. (**A**) PSS-10 total score by treatment. (**B**) IES-R total score by treatment. (**C**) GAD-7 total score by treatment. *Notes*: ***p < 0.01, **p < 0.05, and *p < 0.10; mean comparisons are performed between treatments and the *No extension* baseline using *t*-tests; scores for the *Two extension* treatment correspond to those elicited after the second 2-week extension.

Figure 2 plots the total score of the mental health measures by treatment. Panel A displays the PSS-10 total score, with higher scores indicating greater perceived distress/stress feelings. The mean PSS-10 total score is 16.90 (s.d. = 7.22, range: 0–40), which is higher than that reported in the general population under normal circumstances (< 13 according to^56^) and slightly lower than the values reported by^63^ during the SARS outbreak in 2003 (18.5; |*t|*= 4.26, *p* = 0.000), and^9^ during the COVID-19 pandemic (17.4; |*t|*= 1.76, *p* = 0.078). When looking at treatment effects, results from unpaired *t*-tests show that participants in the *Two extensions* treatment exhibit higher distress levels compared to those in the *One extension* and *No extension* treatments (|*t|*= 3.39, *p* < 0.001, Cohen’s d = 0.228, and |*t|*= 3.35, *p* < 0.001, Cohen’s d = 0.228, respectively). No significant effects are found for the *One extension* treatment compared to the *No extension* baseline (|*t|*= 0.08, *p* = 0.938, Cohen’s d = 0.006). Regarding individuals’ anxiety level, the mean GAD-7 total score is 6.56 (s.d. = 6.15, range: 0–21), which is indicative of a mild anxiety level^58^. Similar to the PSS-10 results, subjects in the *Two extensions* treatment exhibit higher levels of anxiety compared to those in the *One extension* and *No extension* treatments (|*t|*= 3.53, *p* < 0.001, Cohen’s d = 0.233, and |*t|*= 2.13, *p* = 0.033, Cohen’s d = 0.142, respectively) (Panel C). In contrast to distress and anxiety, no significant treatment effects are found for PTSD symptoms (*p* > 0.10 in all *t*-tests) (Panel B). [|*t|*= 0.53, *p* = 0.599, Cohen’s d = 0.035, and |*t|*= 0.30 for *Two extensions* treatment compared to *One extension* treatment; *p* = 0.768, Cohen’s d = 0.020 for *Two extensions* treatment compared to *No extension*.] Importantly, previous studies have reported higher IES-R scores for longer quarantine periods^27,28^, and a positive association between PTSD symptoms and increased exposure and constant reminders to the scenes of trauma (e.g., wars and epidemics)^30,60^. Therefore, we can potentially attribute the absence of a treatment effect on PTSD to the high level of uncertainty about the coronavirus during its initial stage and the fact that respondents had been exposed to a relatively short quarantine period when the survey was implemented. The mean IES-R score is 29.91 (s.d. = 21.57, range: 0–88), which aligns with the finding by^22^ of a 32.98 mean score among Chinese adults during the first weeks of the COVID-19 outbreak. A cut-point of 20 indicates the prevalence of PTSD symptoms and is used to enable comparisons with previous studies^27,28^. An IES-R score of at least 20 is found for approximately 63% of respondents. The mean IES-R score and percentage of subjects with a score of ≥ 20 in our sample are higher than values reported by^28^ during SARS. The significantly higher mental health scores (i.e. PSS-10 and GAD-7) exhibited by subjects in the Two *extensions* treatment compared to those in the *One extension* condition indicates that providing two extensions in shorter “chunks” of time causes more stress and anxiety than providing a one-time extension equivalent to the same length of time. [A potential concern with the validity of our findings relates to the hypothetical nature of the extension scenarios (manipulations). We conjecture that the significant treatment effects found on subjects’ stress and anxiety levels could be considered as lower bounds. That is, the treatment effects are more likely to be higher under real stay-at-home order extensions. In this regard, previous studies have shown that hypothetical scenarios do impact the mental capacity of individuals^32,33^.] This result suggests that the number of extensions and how those are implemented and announced, rather than the total duration of the stay-at-home order, matters when implementing policies that aimed to minimize the negative experience of quarantining and its impact on individuals’ mental health. However, these results should be taken with some precaution as it is possible that the significant effect observed in the *Two extension*s treatment is simply a consequence of making the order extension more salient by providing subjects with two stay-at-home orders rather than only one in the other treatment. To minimize the potential for this issue, we implemented a between-subject design where participants were only assigned to one treatment arm. In addition, the procedure implemented in our experiment would be similar to the implementation of actual directives in a real-world setting. In fact, the way stay-at-home order extensions were mandated and announced in the US varied across states. For example, Indiana’s stay-at-home order, originally supposed to end on April 6, was first extended by 2 weeks until April 20, followed by another 2-week extension through May 1 of 2020. Similarly, consecutive order extensions for short periods were mandated in states like Washington, Michigan, New Mexico, and Massachusetts. In contrast, authorities from Georgia and New Hampshire announced a single (or fewer) order extension but lasting longer periods of time^31^.

Recall that subjects’ expectations of stay-at-home order extensions were assessed before they received the additional stay-at-home extension treatment manipulation. This allows us to answer our second research question: How do prior expectations to stay-at-home order extensions affect individuals’ mental wellbeing and their response to treatments? To answer this question, subjects are asked to imagine a scenario where a 4-week stay-at-home order has taken place in the state they reside. They are then asked whether they expect an extension to this stay-at-home order, and if so, by how many more weeks. To align the responses with the treatments, we split prior expectations (i.e., expectations pre-treatment) to stay-at-home order extensions into 2 categories: expected extension of 4 weeks or less and expected extension of more than 4 weeks. This means that for all treatments, subjects with extension expectations of more than 4 weeks overestimate the actual extension received in the treatment. S1 Table displays the sample size by expected extension category and treatment. The distribution of expected extensions is balanced across treatments, with a larger number of subjects expecting an extension of 4 weeks or less in all treatments.

**Result 2.** *Expecting stay-at-home order extensions of more than 4 weeks increases individuals’ distress, anxiety, and PTSD symptoms.*

**Figure 3:**
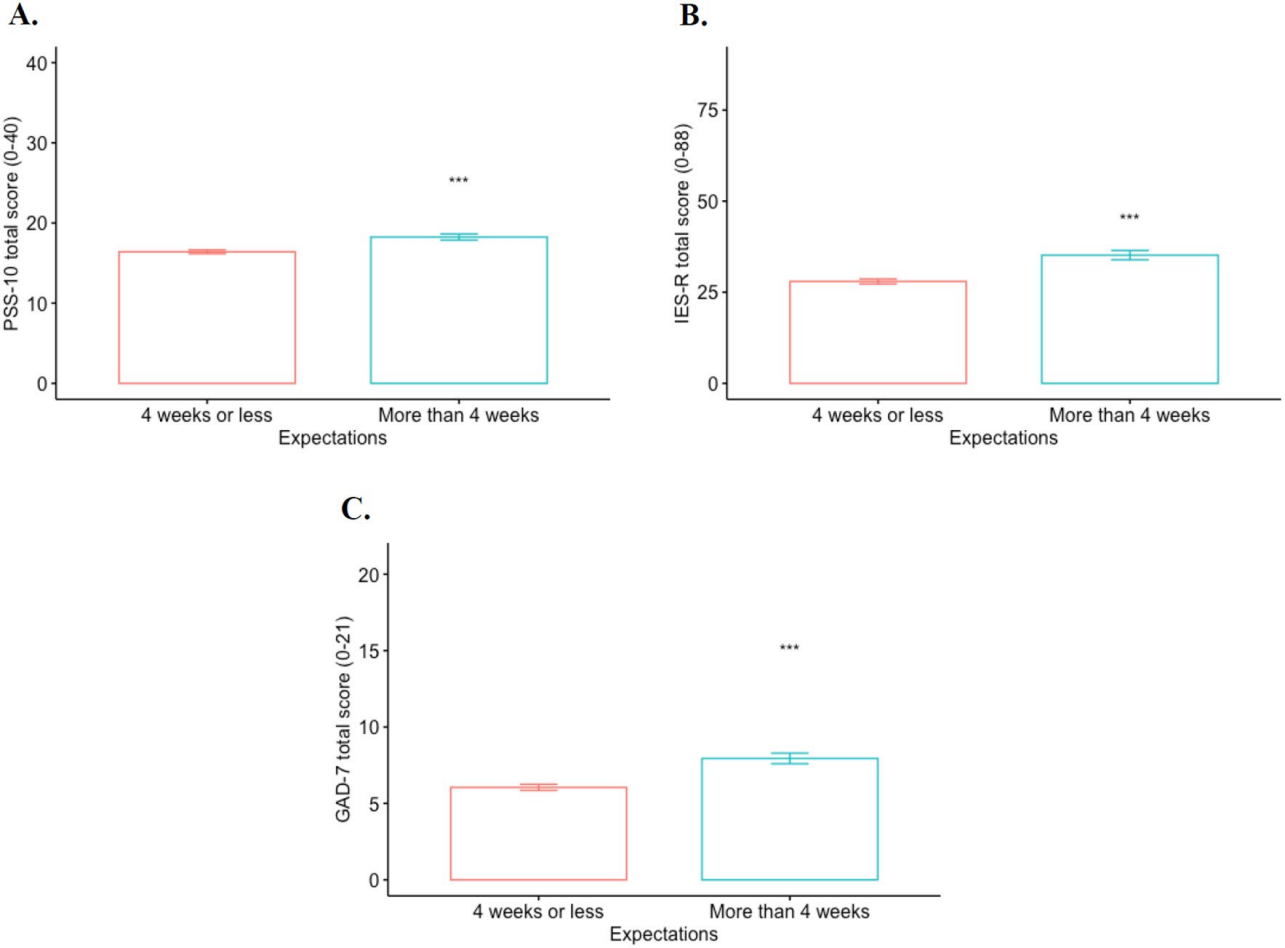
Psychological total scores by expectation category. (**A**) PSS-10 total score by expectation category. (**B**) IES-R total score by expectation category. (**C**) GAD-7 total score by expectation category. ***p < 0.01, **p < 0.05, and *p < 0.10; mean comparisons between two categories are performed using *t*-tests; scores for the *Two extension* treatment correspond to those elicited after the second 2-week extension.

Figure 3 displays the total score for the mental health measures by expectation category. The results show that subjects who expected an order extension of more than four weeks exhibit higher levels of distress (Panel A), PTSD symptoms (Panel B), and anxiety (Panel C) compared to subjects with expectations of less than four weeks (|*t|*> 2.58, *p* < 0.001 for all *t*-tests). [Cohen’s d = 0.256, 0.338, and 0.311 for comparisons in Panel A, Panel B and Panel C, respectively.] This means that the longer the prior expectations on the extension duration, the greater the harm to individuals’ mental wellbeing. This aligns with evidence suggesting that increasingly long projections for self-quarantining can worsen individuals’ experience of isolation^30^, which also decreases their likelihood to comply with directives^29^. It is possible that the uncertainty in itself may increase the level of psychological and emotional damage.

More formally, we investigate the treatment effects on the intensity of the mental health measures using OLS specifications and present the results in Table 2. The dependent variables correspond to the PSS-10 score for distress in specifications (1)–(3), IES-R score for PTSD in specifications (4)–(6), and GAD-7 for anxiety in specifications (7)–(9). The results from Table 2 show that subjects who received two 2-week stay-at-home order extensions exhibit higher levels of distress and anxiety compared to those who received no extension. When comparing the regression coefficients between the *Two extensions* treatment and *One extension* treatment using F-tests, we find significant differences in the distress and anxiety levels between treatments in all specifications (with and without controls), which is consistent with the results from Fig. 1 (*F* = 11.52, *p* < 0.001 in (1), and *F* = 6.30, *p* = 0.012 in (3) for PSS-10; *F* = 12.44, *p* < 0.001 in (7), and *F* = 9.55, *p* = 0.002 in (9) for GAD-7). Moreover, participants who expected extensions of more than four weeks exhibited higher distress, anxiety and PTSD symptoms compared to those who expected less than a 4-week extension.

When looking at the individuals’ socio-demographic characteristics, we find that male and older subjects present lower levels of stress and anxiety compared to female and younger subjects, respectively. The gender effect supports previous studies showing that compared to males, females have suffered higher psychological distress as result of the pandemic^19,20,22,23^. On the contrary, the lower anxiety exhibited among the elderly does not align with previous findings reporting a negative psychological effect of binding curfew orders for adults aged 65 and older^43^. Albeit speculative, three factors may explain the low stress level in older subjects in our sample: a limited exposure to the pandemic due to home quarantine, exposure to lower amount of information from social media that can easily trigger stress, and an increased psychological self-regulation^19,23^. We also find that subjects living in rural areas and those affiliated to ‘other’ political affiliation (i.e., other than Republican or Democrat) exhibit lower intensity of PTSD symptoms and anxiety compared to subjects living in urban areas and those affiliated to the Democratic Party. Furthermore, lower psychological distress is found among African-Americans compared to White subjects, and among the high-income group. The opposite effect (i.e., higher anxiety and PTSD symptoms) is found among participants living with an elder and those reporting higher risk seeking behavior.[Risk seeking behavior was measured by asking subjects the question: “How do you see yourself: Are you generally a person who is fully prepared to take risks or do you try to avoid taking risks?” (11-point scale; 0 = Not at all willing to take risks, and 10 = Very willing to take risks).] A higher distress level is expected for household members living with an elder (> 65 years old) due to a relatively higher morbidity rate and risk for depression and mental illness among this age group. In fact, recent evidence suggests that limited social and physical interactions due to the pandemic has acted as an additional stressor, increasing the likelihood of older adults feeling lonelier, more anxious, and forgotten^12,64,65^. Moreover, results from a poll conducted by the American Psychiatric Association (2020) show that about 62% of Americans are anxious and stressed about the possibility of family and loved ones getting coronavirus. We also asked participants whether the presented stay-at-home order extension scenarios seemed sufficient to prevent the disease spread and justifiable given the impact on the economy and employment. While subjects who perceive the extension to be sufficient in preventing the spread of the disease exhibit higher anxiety and PTSD symptoms, those who think the hypothetical extension is justifiable appeared to be less psychologically affected. To examine the conflict between public health and the economy during the pandemic^66^, elicit social distancing and stay-at-home decisions in response to messaging treatments highlighting the health and economic risks of COVID-19. They find heterogeneous responses to the health information messaging based on political partnership, which echoes previous findings by^67,68^. Finally, when controlling for subjects’ feelings of worry towards the pandemic (see estimates in S3 Table), we find experienced worry to be significantly associated with higher distress, anxiety, and PTSD symptoms. That is, those who worried more about the pandemic in the four weeks prior to the study, appeared to be more psychologically affected.

To get a better understanding as to how the hypothetical stay-at-home order extensions affect individuals’ mental health given their prior expectations, we estimate OLS regressions on the intensity of distress, anxiety, and PTSD symptoms by expectation category.

**Result 3.** *There are heterogeneous treatment effects on mental wellbeing across subjects with different prior expectations.*

Table 3 shows the results of OLS regressions on the mental health scores by extension expectation category. Model specifications 1, 3, and 5 correspond to mental health measure estimates of subjects with extension expectations of four weeks or less. Model specifications 2, 4, and 6 correspond to estimates of subjects with expected extensions of more than four weeks. [Results from Chow tests suggest that separate regressions on the expectation categories delivers a better model than a combined (pooled) regression; *F* = 1.71, *p* = 0.036 for PSS-10; *F* = 3.39, *p* < 0.001 for IES-R; *F* = 2.44, *p* < 0.001for GAD-7.] When looking at subjects with extension expectations of four weeks or less, we find a significant positive effect of the *Two extensions* treatment on the PSS-10 and GAD-7 total scores. That is, subjects who underestimate the extension duration of the stay-at-home order exhibit higher distress and anxiety under the 2-week extensions compared to no extension. On the contrary, no treatment effects are found for individuals with expected extensions of more than four weeks. This means that when participants have a longer expectation for the duration of the stay-at-home order, their mental wellbeing is not affected by the way the extensions are announced in the treatments.

Regarding socio-economic characteristics, we find that males and older subjects with expected extensions of 4 weeks or less suffer less psychological distress compared to females and young subjects within the same expectation category. Similarly, lower scores in all mental health measures are reported among subjects within the same expectation category who perceive the extension as being justifiable and those affiliated to ‘other’ political party. For individuals with expected extensions of more than four weeks, we find that those who perceive the stay-at-home order extension as sufficient and those with more risk seeking behavior present higher levels of anxiety and PTSD symptoms. Importantly, the actual lockdown duration at the time of the survey does not seem to affect subjects’ mental health, suggesting that the observed effects are mainly driven by the treatments.Finally, among subjects with expected extensions of four weeks or less, we find that those with a higher level of experienced worry and a higher perceived likelihood of getting infected exhibit higher distress and anxiety. Similarly, higher scores of distress and PTSD symptoms are observed among subjects with higher experienced worry and perceived absolute susceptibility in the opposite expectation category.

Our findings show that the treatment effects on mental wellbeing depend on the prior expectations of individuals. In particular, the announcement of two shorter extensions negatively impacts the mental health only of individuals with expectations of four weeks or less. Moreover, we find heterogeneous effects of individual factors (i.e., socio-demographics) on psychological states across subjects with different prior extension expectations. These findings highlight the importance of the type of information that is conveyed by news outlets and the media in terms of stay-at-home extension orders, as it may play a key role in forming public’s expectations.

## Discussion and implications

Stay-at-home or lockdown orders can cause mental health problems. In this paper, we examine whether hypothetical scenarios for stay-at-home order extensions and the way they are implemented (i.e., whether in multiple chunks of time or one single order for the same period) influence mental health outcomes. This is an important topic since it can help inform government or public health officials on how to present or frame stay-at-home orders that can minimize mental health problems. Given that it is hard to find observational data and challenging to use such data to estimate the causal effect of stay-at-home orders, we designed a study that exogenously assigned subjects to different hypothetical stay-at-home extension order scenarios. Our results suggest that the mental health outcomes can depend on the number of stay-at-home order extensions and the way these are announced—either as a longer one-time extension or two shorter extensions. Specifically, we find that exposing participants to two consecutive 2-week stay-at-home order extensions produces higher level of stress and anxiety compared to those receiving one 4-week extension or no extension. It is important to note that although the observed treatments effects on mental health outcomes are in the range of what is considered a small effect size (i.e. Cohen’s d ranging from 0.142 to 0.338 for significant results), these can still be seen as lower bounds of the high impact that the coronavirus imposed on people’s mental wellbeing globally and provide insights on how to manage extensions when lockdown mandates are required. It is notable to find effects with our interventions since the mental health impacts were already quite large because of exposure to the largest pandemic of our generation. Our findings suggest that when coping with COVID-19 directives, individuals may prefer to receive a one-time longer isolation period. That is, imposing a longer mandate that can be terminated if needed might have some positive effects on mental health.

When looking at subjects’ prior expectations on order extensions, we find that subjects with longer expected extensions exhibit more signs of psychological damage than those with shorter expected extensions. This supports the findings by^49^ suggests that expecting longer periods of self-quarantining due to the coronavirus may actually worsen the experience of isolation. For example, depression and anxiety levels seem to increase as the time spent in lockdown progresses^48^. Furthermore, we find that subjects’ expectations on extension duration influence the treatment effects on mental health. In particular, the negative psychological consequences of providing two shorter extensions are observed only among subjects with expectations of four weeks or less. This is important as it demonstrates that people’s expectations affect the level of psychological damage caused by lockdown mandates.

In conclusion, our study provides insights on the psychological impact of lockdown extensions and suggests ways as to how these extensions can be implemented and publicly announced to minimize mental health problems. If lockdown extensions are essential, then we recommend that the mandated extension is provided as a one-time extension, rather than in multiple smaller extensions. Moreover, public announcements and the general attitudes of government officials are crucial for generating people’s expectations on lockdown time duration which may impact their mental wellbeing and their willingness to comply with home quarantine and social isolation. This highlights the importance of transparency, good information governance, and leadership across regulatory authorities to reduce the mental health and psychosocial burden of a pandemic. The results from this study open the door to a new research agenda. The psychological well-being of quarantined individuals, their responses to directives and the outcomes of intervention programs need to be set as priority areas for future research. This is timely research as many countries have started to react to future waves of the pandemic without a long-term plan and they may face an exhausting series of lockdowns that could substantially affect mental health and adherence to directives.

## Limitations

The limitations of this study are related to the survey construction and sampling method. First, our treatment scenarios are hypothetical since stay-at-home order durations and lockdown extensions varied by state at the time of the survey. To help mitigate hypothetical bias in our results, we implemented a COVID-19 related cheap talk and constant reminders of the importance of truthful reporting. Second, there are several other measures regarding psychological well-being that could have been used to explore the motivations and results. We selected three validated measures of stress, anxiety, and PTSD symptoms that have been previously used to assess the effects of quarantining on mental health. Finally, we acknowledge that although this survey was conducted using a specific group of respondents (i.e., Amazon M-Turk Workers), the use of the online platform allowed us to reach a wide geographical representation across the US, and it was the most feasible way to collect data to obtain a diverse representation. Despite these shortcomings, we believe that this study provides important insights about the impact of lockdown extensions on mental health, particularly in situations of high uncertainty, and how these extensions can be implemented and publicly announced to minimize the potential negative psychological consequences.

### Supplementary Information

Supplementary Information.

## Data Availability

The datasets generated and analyzed during the current study are available from the corresponding author on reasonable request.
