# Supplementary material for: The mental burden of stay-at-home order extensions during COVID-19
Source: Sci Rep. 2024 Feb 21;14:4293. doi: 10.1038/s41598-024-54059-z (PMC10881574; doi:10.1038/s41598-024-54059-z)
Supplement: Supplementary file 1 — Supplementary Information. [file 41598_2024_54059_MOESM1_ESM.docx]

**The Mental Burden of Stay-At-Home Order Extensions during COVID-19**

**S1 Appendix**

**Cheap talk**

“Studies show that people tend to act differently when they face hypothetical decisions. In other words, they say one thing and do something different. For example, some people would state they are willing to comply with all the COVID-19 guidelines and restrictions, but when this action becomes available to them, they will fail to adhere to the directives in the way they said they would. There can be several reasons for this behavior. It might be that it is too difficult to measure the impact of one's compliance in the overall spread of the pandemic. Another possibility is that it might be difficult to visualize the consequences of not adhering to the mandates in a hypothetical scenario. We want you to behave in the same way that you would if you really had to choose whether to adhere to the COVID-19 regulations or not. Please keep this in mind when answering the following questions.”

**Treatment Scenarios**

For each of the three conditions, subjects first received the following pandemic-related description: “A vast majority of states in the US have instructed residents to stay at home as a measure to prevent the spread of the coronavirus (COVID-19). The stay-at-home order directs all residents to avoid leaving their homes unless necessary and to practice social distancing and wear mask, among others, when they need to travel outside their homes to work, access foods, prescriptions, health care, and other necessities. Outdoor exercise is allowed as long as people maintain six-foot physical distancing. The end dates for the stay-at-home order vary by state, with the possibility of them being restricted or extended.”

*Control*: “Imagine that a 4-week stay-at home order has taken place in the state that you reside.”

*One-long extension* treatment: “For the next seven questions, please imagine that a 4-week stay-at-home order has taken place in the state you reside, but the order has been re-evaluated before its expiration date and the governor has decided to issue an extension for another **four weeks (now for a total of a 8-week stay-at-home order)**.”

*Two-short extensions* treatment – first extension: “For the next seven questions, please imagine that a 4-week stay-at-home order has taken place in the state you reside, but the order has been re-evaluated before its expiration date and the governor has decided to issue an extension for another **two weeks (now for a total of a 6-week stay-at-home order)**.”

*Two-short extensions* treatment – second extension: “For the next seven questions, please imagine that the 2-week stay-at-home order extension has been further extended for an additional **two weeks (now for a total of an 8-week stay-at-home order).**

The next seven questions are similar to those you already responded to earlier but we are asking these questions again for this second **2-week extension scenario** to know if there are changes in how you will behave and feel about it. Please provide us with truthful answers to all the questions.”

**S2 Appendix**

**Experimental Instructions**

**Informed Consent: Please indicate that you are 18 years and above, you are aware that your answers will remain anonymous and your participation in this survey is completely voluntary.**

1. Yes [Proceed to Survey]
2. No

Do you live in a state with a stay-at-home order?

- 1. Yes [Proceed to Survey]
  2. No

We care about the quality of our survey data and hope to receive the most accurate measures of your opinions. Therefore, it is important that you **carefully read all the information provided, and that you thoughtfully give your best answer to each question in the survey**.

Do you commit to carefully reading and providing your thoughtful and honest answers to the questions in this survey?

1. I will read carefully and provide my best answers [Proceed to Survey]
2. I will not read carefully and provide my best answers
3. I can’t promise either way

The goal of the survey is to understand how a pandemic like the novel coronavirus affects people's behavior. The findings can help policy makers, communities and companies in emergency/crisis management efforts.

Studies show that people tend to act differently when they face hypothetical decisions. In other words, they say one thing and do something different. For example, some people would state they are willing to comply with all the COVID-19 guidelines and restrictions, but when this action becomes available to them, they will fail to adhere to the directives in the way they said they would. There can be several reasons for this behavior. It might be that it is too difficult to measure the impact of one’s compliance in the overall spread of the pandemic. Another possibility is that it might be difficult to visualize the consequences of not adhering to the mandates in a hypothetical scenario. We want you to behave in the same way that you would if you really had to choose whether to adhere to the COVID-19 regulations or not. Please keep this in mind when answering the following questions.

1. To what extent have you been following the stay-at-home order of your state? (0= not at all; 1= a little bit; 2= moderately; 3=quite a bit; 4=extremely)
2. Which of these directives are you currently following? Please mark Yes or No to each of the following statements.
   1. Stay home from work.
   2. Use drive-thru, pickup, or delivery options.
   3. Avoid discretionary travel, shopping trips, and social visits.
   4. Avoid visiting nursing homes or retirement facilities unless to provide critical assistance.
   5. Avoid social gatherings in groups of more than 10 people.
   6. Wear cloth face coverings (masks) in public settings.

A vast majority of states in the US have instructed residents to stay at home as a measure to prevent the spread of the coronavirus (COVID-19). The stay-at-home order directs all residents to avoid leaving their homes unless necessary and to practice social distancing and wear mask, among others, when they need to travel outside their homes to work, access foods, prescriptions, health care, and other necessities. Outdoor exercise is allowed as long as people maintain six-foot physical distancing. The end dates for the stay-at-home order vary by state, with the possibility of them being restricted or extended.

*For no extension or control:* Imagine that a 4-week stay-at-home order has taken place in the state you reside, would you expect your state government to extend this stay-at-home order? If yes, by how many more weeks?

*For One extension treatment:* For the next seven questions, please imagine that a 4-week stay-at-home order has taken place in the state you reside, but the order has been re-evaluated before its expiration date and the governor has decided to issue an extension for another **four weeks (now for a total of 8-week stay-at-home order)**.

*For Two extensions treatment:* For the next seven questions, please imagine that a 2-week stay-at-home order has taken place in the state you reside, but the order has been re-evaluated before its expiration date and the governor has decided to issue an extension for another **four weeks (now for a total of 6-week stay-at-home order)**.

***Note:*** the following questions are for the control condition. For the treatments the stay-at-home order extension was modified accordingly.

1. With this **4-week stay-at-home order,** would you be willing to help control the spread of the disease by adhering to each of the following directives? (Yes; No; Unsure)
   1. Stay home from work.
   2. Use drive-thru, pickup, or delivery options.
   3. Avoid discretionary travel, shopping trips, and social visits.
   4. Avoid visiting nursing homes or retirement facilities unless to provide critical assistance.
   5. Avoid social gatherings in groups of more than 10 people.
   6. Wear cloth face coverings (masks) in public settings.
2. Do you think a **4-week stay-at-home order** would be sufficient in preventing the spread of the disease?
   1. Yes
   2. No
3. Do you think a **4-week stay-at-home order** is justifiable given the impact on the economy and employment?
   1. Yes
   2. No
4. With this **4-week stay-at-home order,** would you expect more lockdowns or stay at home orders in the next two months?
   1. Yes
   2. No
5. With this **4-week stay-at-home order**, please rate how often do you think the following statements will happen to you (0= never; 1= almost never; 2= sometimes; 3=fairly often; 4=very often)
6. Be upset because of something that happened unexpectedly.
7. Feel that I am unable to control the important things in my life.
8. Feel nervous and “stressed”.
9. Feel that things are going my way.
10. Feel confident about my ability to handle my personal problems.
11. Find that I cannot cope with all the things that I have to.
12. Be able to control irritations in my life.
13. Feel that I am on top of things.
14. Be angered because of things that are outside of my control.
15. Feel difficulties will be piling up so high that I cannot overcome them.
16. Below is a list of difficulties people sometimes have after stressful life events. Please read each item, and then indicate how you might react **after the 4-week stay-at-home order** with respect to your COVID-19 quarantine experience. (0= not at all; 1= a little bit; 2= moderately; 3=quite a bit; 4=extremely)
17. Any reminder would bring back feelings about it.
18. I would have trouble staying asleep.
19. Other things would keep me thinking about it.
20. I would feel irritable and angry.
21. I would avoid letting myself get upset when I think about it or are reminded about it.
22. I would think about it when I do not mean to.
23. I would feel as if it has not happened or is not real.
24. I would stay away from reminders of it.
25. Pictures about it would pop into my mind.
26. I would feel jumpy and easily startled.
27. I would try not to think about it.
28. I would be aware that I still have many feelings about it, but I do not deal with them.
29. My feelings about it would kind of numb.
30. I would find myself acting or feeling like I am back at that time.
31. I would have trouble falling asleep.
32. I would have waves of strong feelings about it.
33. I would try to remove it from my memory.
34. I would have trouble concentrating.
35. Reminders of it would cause me to have physical reactions, such as sweating, trouble breathing, nausea, or a pounding heart.
36. I would have dreams about it.
37. I would feel watchful and on-guard.
38. I would try not to talk about it.
39. With this **4-week extension** **stay-at-home order**, how often would you be bothered by the following problems? (0= not at all; 1= several days; 2= more than half the days; 3= nearly every day)
    1. Feeling nervous, anxious or on the edge.
    2. Not being able to stop or control worrying.
    3. Worrying too much about different things.
    4. Trouble relaxing.
    5. Being so restless that it is hard to sit still.
    6. Becoming easily annoyed or irritable.

Feeling afraid as if something awful might happen.

*For the Two extensions treatment only:* For the next seven questions, please imagine that the 2-week stay-at-home order extension has been further extended for an additional **two weeks (now for a total of 8-week stay-at-home order).**

The next seven questions are similar to those you already responded to earlier but we are asking these questions again for this second 2-week extension scenario to know if there are changes in how you will behave and feel about it. Please provide us with truthful answers to all the questions. (Repeat previous seven questions (3-9) but now with additional 2-week extension).

***Note****:* Subjects in all conditions completed the following questions.

The following questions do not pertain to the hypothetical scenario described above, but rather to your **actual COVID-19 experience**. Please respond appropriately and truthfully to each question. Remember that your responses will be kept confidential.

1. In the past 4 weeks, have your ever worried about catching COVID-19? (1= no, never think about it; 5= worried about it all the time)
2. Please rate the current level of your worry towards COVID-19. (1= very mild; 7= very severe)
3. If you were to develop COVID like symptoms tomorrow, would you be: (1= not at all worried; 7=extremely worried)
4. How likely do you think it is that you will contact COVID-19 over the next month? (1= never; 7= certain)
5. What do you think are your chances of getting COVID-19 over the next month compared to others outside your family? (1= not at all; 7=certain)
6. In what state do you currently reside? (at the moment of the survey)
7. Do you expect an extension to the stay-at-home-order of your state?
   1. Yes
   2. No
8. How many weeks (in total) of stay at home order would you find reasonable given the tradeoff between public health and the health of the economy?
9. Would you expect a second wave of COVID-19 infections if stay-at-home orders are lifted sooner than what public health experts recommend?
   1. Yes
   2. No
10. If your state government ends the stay-at-home order today and allows the opening of gyms and restaurants, would you go to a restaurant or gym within a week of ending the lock-down?
    1. Yes
    2. No
11. How would you describe your residential area?
    1. Urban
    2. Suburban
    3. Rural
12. What has your isolation environment been since the stay-at-home order issuance?
    1. Hospital
    2. Alone (at home or hotel)
    3. With family or friends
13. What social network activities are you performing since the state-at-home order issuance?
    1. Making phone calls
    2. Texting or e-mailing
    3. Using the internet
    4. None
    5. Other (please specify)
14. Have you experienced COVID-19 like symptoms?
    1. Yes
    2. No
15. Have you incurred medical expenditures due to COVID-19?
    1. Yes
    2. No
16. Have you incurred financial losses due to COVID-19? (e.g., business closure, debt repayment defaults, reduction in return of investments—stock market, real state).
    1. Yes
    2. No
17. Have you lost your job due to COVID-19?
    1. Yes
    2. No
18. Have you experienced new financial opportunities due to COVID-19? (e.g., invest in stock market, participate in delivery services).
    1. Yes
    2. No
19. What is your gender?
    1. Male
    2. Female
    3. Other
20. What is your age?
21. Including yourself, how many people live in your household?
22. Do you have children currently living in your household?
    1. Yes
    2. No
23. Do you have elders (65+ years old) currently living with you?
    1. Yes
    2. No
24. Do you have pets?
    1. Yes
    2. No
25. What is your ethnicity?
26. Caucasian
27. African American
28. Native American
29. Hispanic/ Latino
30. Asian/ Pacific Islander
31. Other (please specify)
32. What is the highest level of education that you obtained?
    1. Some high school or less
    2. High School diploma
    3. Some college
    4. 2 year/Associates degree
    5. 4 year/Bachelor’s degree
    6. Some graduate school
    7. Graduate school
33. What is your employment status?
    1. Full-time employed
    2. Part-time employed
    3. Unemployed
    4. Retired
    5. Homemaker
    6. Student
    7. Other
34. Are you a healthcare professional?
    1. Yes
    2. No
35. What was your 2019 household income before taxes?
36. Less than $30,000
37. $30,000-$39,999
38. $40,000-$49,999
39. $50,000-$59,999
40. $60,000-$69,999
41. $70,000-$79,999
42. $80,000-$89,999
43. $90,000-$99,999
44. $100,000-$149,999
45. $150,000 or more
46. What is your political affiliation?
47. Republican party
48. Democratic party
49. Other (please specify)
50. None
51. On a scale from 1 to 10, where 1 is not at all willing to take a risk and 10 is very willing to take risks, how would you rate yourself?

| **Not all willing to take risks** | | |  |  |  |  | **Very willing to take risks** | | |
| --- | --- | --- | --- | --- | --- | --- | --- | --- | --- |
| 1 | 2 | 3 | 4 | 5 | 6 | 7 | 8 | 9 | 10 |

1. Do you have any comments? (Optional)

**S3 Appendix**

**Additional Analysis**

**S1 Table.** Sample size by expectation category and treatment.

| **Expected extension** |  | **Treatments** |  |
| --- | --- | --- | --- |
|  | No extension | One extension | Two extensions |
| 4 weeks or less | 274 | 265 | 381 |
| More than 4 weeks | 90 | 95 | 154 |

**S2 Table.** OLS regressions on psychological measures - first extension for Two extensions treatment.

|  | PSS-10 (1) | PSS-10 (2) | PSS-10 (3) | IES-R (4) | IES-R (5) | IES-R (6) | GAD-7 (7) | GAD-7 (8) | GAD-7 (9) |  |
| --- | --- | --- | --- | --- | --- | --- | --- | --- | --- | --- |
|  |  |  |  |  |  |  |  |  |  |  |
| One Extension | 0.041 | 0.013 | -0.081 | -0.308 | -0.431 | -0.706 | -0.544 | -0.573 | -0.546 |  |
|  | (0.511) | (0.508) | (0.486) | (1.535) | (1.516) | (1.346) | (0.441) | (0.438) | (0.398) |  |
| Two Extensions | 1.549*** | 1.479*** | 1.190** | 1.604 | 1.302 | 2.287* | 0.419 | 0.346 | 0.472 |  |
|  | (0.467) | (0.464) | (0.484) | (1.403) | (1.386) | (1.342) | (0.403) | (0.400) | (0.396) |  |
| Expect more than 4 w extension |  | 1.707*** | 1.321*** |  | 7.433*** | 5.689*** |  | 1.797*** | 1.454*** |  |
|  |  | (0.434) | (0.462) |  | (1.296) | (1.280) |  | (0.374) | (0.378) |  |
| Justifiable |  |  | -1.729*** |  |  | -2.382* |  |  | -0.624 |  |
|  |  |  | (0.472) |  |  | (1.307) |  |  | (0.386) |  |
| Sufficient |  |  | 0.858** |  |  | 6.586*** |  |  | 0.948*** |  |
|  |  |  | (0.412) |  |  | (1.140) |  |  | (0.337) |  |
| Rural |  |  | -0.53 |  |  | -3.698** |  |  | -0.905** |  |
|  |  |  | (0.545) |  |  | (1.510) |  |  | (0.446) |  |
| Living with elder |  |  | 0.761 |  |  | 4.301*** |  |  | 1.243*** |  |
|  |  |  | (0.508) |  |  | (1.409) |  |  | (0.416) |  |
| Risk seeking |  |  | 0.043 |  |  | 1.611*** |  |  | 0.277*** |  |
|  |  |  | (0.080) |  |  | (0.222) |  |  | (0.066) |  |
| Other polit. affiliation |  |  | -0.698 |  |  | -5.696*** |  |  | -1.541*** |  |
|  |  |  | (0.517) |  |  | (1.432) |  |  | (0.423) |  |
| Republican affiliation |  |  | -0.611 |  |  | 0.093 |  |  | -0.204 |  |
|  |  |  | (0.484) |  |  | (1.340) |  |  | (0.396) |  |
| Age |  |  | -0.121*** |  |  | -0.347*** |  |  | -0.121*** |  |
|  |  |  | (0.014) |  |  | (0.039) |  |  | (0.012) |  |
| Male |  |  | -1.454*** |  |  | -1.448 |  |  | -0.930*** |  |
|  |  |  | (0.430) |  |  | (1.190) |  |  | (0.352) |  |
| Income |  |  | -0.000 |  |  | 0.000 |  |  | 0.000 |  |
|  |  |  | (0.000) |  |  | (0.000) |  |  | (0.000) |  |
| African American |  |  | -1.463** |  |  | 0.985 |  |  | -0.648 |  |
|  |  |  | (0.594) |  |  | (1.646) |  |  | (0.486) |  |
| Hispanic |  |  | 1.187 |  |  | 3.369** |  |  | -0.002 |  |
|  |  |  | (0.766) |  |  | (2.124) |  |  | (0.627) |  |
| Other race |  |  | 0.236 |  |  | -1.750 |  |  | -0.596 |  |
|  |  |  | (0.694) |  |  | (1.924) |  |  | (0.568) |  |
| Lockdown length |  |  | 0.031 |  |  | -0.068 |  |  | -0.011 |  |
|  |  |  | (0.025) |  |  | (0.071) |  |  | (0.021) |  |
| Constant | 16.184*** | 15.762*** | 22.608*** | 29.808*** | 27.970*** | 37.000*** | 6.332*** | 5.888*** | 11.326*** |  |
|  | (0.360) | (0.374) | (1.389) | (1.082) | (1.116) | (3.850) | (0.311) | (0.332) | (1.137) |  |
| Observations | 1259 | 1259 | 1076 | 1259 | 1259 | 1076 | 1259 | 1259 | 1076 |  |
| R^2^ | 0.012 | 0.024 | 0.154 | 0.002 | 0.027 | 0.256 | 0.004 | 0.022 | 0.21 |  |

*Notes:* ***p<0.01, **p <0.05, and *p<0.10; scores for the *Two extension* treatment correspond to those elicited after the first 2-week extension.

**S3 Table.** OLS regressions on psychological measures with additional controls.

|  | PSS-10 (All sample) (1) | PSS-10 (4 w or less) (2) | PSS-10 (More than 4 w) (3) | IES-R (All sample) (4) | IES-R (4 w or less) (5) | IES-R (More than 4 w) (6) | GAD-7 (All sample) (7) | GAD-7 (4 w or less) (8) | GAD-7 (More than 4 w) (9) |  |
| --- | --- | --- | --- | --- | --- | --- | --- | --- | --- | --- |
|  |  |  |  |  |  |  |  |  |  |  |
| One Extension | 0.02 | -0.12 | -0.09 | -0.06 | 0.07 | -2.13 | -0.46 | -0.48 | -1.00 |  |
|  | (0.47) | (1.55) | (0.99) | (1.25) | (1.42) | (2.83) | (0.36) | (0.41) | (0.80) |  |
| Two Extensions | 0.86* | 0.53 | 1.50 | -1.27 | -1.53 | -1.67 | 0.32 | 0.25 | 0.37 |  |
|  | (0.48) | (0.58) | (0.93) | (1.31) | (1.56) | (2.60) | (0.37) | (0.44) | (0.78) |  |
| Justifiable | -2.67*** | -2.79*** | -2.46** | -5.08*** | -5.55*** | -5.45** | -1.80*** | -2.08*** | -1.06 |  |
|  | (0.47) | (0.53) | (1.01) | (1.20) | (1.38) | (2.64) | (0.35) | (0.41) | (0.74) |  |
| Sufficient | 0.77* | 0.85* | 0.58 | 5.66*** | 4.76*** | 8.42*** | 0.90*** | 0.59 | 1.86*** |  |
|  | (0.42) | (0.49) | (0.82) | (1.09) | (1.26) | (2.42) | (0.32) | (0.37) | (0.70) |  |
| Rural | -0.19 | 0.28 | -1.50 | -2.04 | -1.59 | -3.61 | -0.53 | -0.27 | -1.23 |  |
|  | (0.55) | (0.63) | (1.24) | (1.47) | (1.67) | (3.11) | (0.42) | (0.48) | (0.92) |  |
| Living with elder | 0.46 | 0.45 | 0.33 | 3.16** | 1.86 | 3.58 | 0.78** | 0.61 | 0.79 |  |
|  | (0.51) | (0.62) | (0.91) | (1.35) | (1.61) | (2.58) | (0.39) | (0.47) | (0.69) |  |
| Risk seeking | -0.04 | -0.12 | 0.13 | 1.17*** | 0.94*** | 1.46*** | 0.15** | 0.09 | 0.24* |  |
|  | (0.09) | (0.10) | (0.17) | (0.24) | (0.27) | (0.49) | (0.07) | (0.07) | (0.13) |  |
| Other polit. affiliation | -0.52 | -0.50 | 0.08 | -5.78*** | -6.62*** | -0.18 | -1.36*** | -1.50*** | -0.14 |  |
|  | (0.51) | (0.57) | (1.27) | (1.32) | (1.49) | (3.16) | (0.38) | (0.43) | (0.93) |  |
| Republican affiliation | -0.23 | 0.20 | -1.45* | 1.40 | 0.82 | 3.35 | 0.46 | 0.71* | -0.18 |  |
|  | (0.46) | (0.54) | (0.81) | (1.26) | (1.47) | (2.45) | (0.36) | (0.42) | (0.70) |  |
| Age | -0.11*** | -0.11*** | -0.12*** | -0.27*** | -0.21*** | -0.35*** | -0.10*** | -0.09*** | -0.11*** |  |
|  | (0.01) | (0.02) | (0.03) | (0.04) | (0.05) | (0.07) | (0.01) | (0.01) | (0.02) |  |
| Male | -1.30*** | -1.39*** | -1.18 | -0.72 | -0.86 | -0.83 | -0.59* | -0.55 | -0.75 |  |
|  | (0.42) | (0.50) | (0.75) | (1.18) | (1.37) | (2.24) | (0.33) | (0.39) | (0.60) |  |
| Income | -0.00** | -0.00 | -0.00*** | -0.00 | -0.00 | 0.00 | 0.00 | 0.00 | -0.00 |  |
|  | (0.00) | (0.00) | (0.00) | (0.00) | (0.00) | (0.00) | (0.00) | (0.00) | (0.00) |  |
| African American | -1.10* | -1.37** | -0.97 | 2.94* | 4.62** | -2.05 | 0.10 | 0.29 | -0.88 |  |
|  | (0.59) | (1.67) | (1.14) | (1.58) | (1.88) | (2.90) | (0.46) | (0.52) | (0.86) |  |
| Hispanic | 0.64 | 0.96 | -0.09 | 3.36* | 4.80** | 0.90 | 0.11 | 0.74 | -1.23 |  |
|  | (0.71) | (0.95) | (1.16) | (1.93) | (2.35) | (3.55) | (0.57) | (0.73) | (0.96) |  |
| Other race | 0.28 | -0.03 | 0.74 | -0.15 | -0.47 | -0.41 | -0.39 | -0.40 | -0.99 |  |
|  | (0.67) | (0.76) | (1.44) | (1.67) | (1.83) | (3.93) | (0.50) | (0.52) | (1.29) |  |
| Lockdown length | 0.04* | 0.03 | 0.04 | -0.03 | -0.01 | -0.11 | -0.01 | -0.01 | -0.03 |  |
|  | (0.02) | (0.03) | (0.03) | (0.06) | (0.08) | (0.11) | (0.02) | (0.02) | (0.03) |  |
| Experienced worry | 0.95*** | 0.98*** | 0.84* | 3.85*** | 4.61*** | 0.92 | 1.30*** | 1.37*** | 1.12** |  |
|  | (0.28) | (0.33) | (0.50) | (0.77) | (0.87) | (1.65) | (0.22) | (0.25) | (0.43) |  |
| Current worry | 0.27 | 0.37* | -0.17 | 1.06** | 0.89 | 1.40 | 0.37*** | 0.31* | 0.37 |  |
|  | (0.19) | (0.22) | (0.33) | (0.51) | (0.58) | (0.98) | (0.14) | (0.16) | (0.27) |  |
| Anticipated worry | 0.29 | 0.16 | 0.61* | -0.95** | -0.99* | -0.59 | -0.07 | -0.01 | -0.10 |  |
|  | (0.20) | (0.25) | (0.34) | (0.48) | (0.58) | (0.87) | (0.14) | (0.17) | (0.25) |  |
| Absolute susceptibility | 0.39* | 0.32 | 0.59 | 2.97*** | 2.57*** | 3.80*** | 0.71*** | 0.87*** | 0.28 |  |
|  | (0.22) | (0.26) | (0.39) | (0.65) | (0.76) | (1.43) | (0.18) | (0.21) | (0.37) |  |
| Relative susceptibility | 0.24 | 0.27 | 0.22 | 0.92 | 0.69 | 1.46 | 0.08 | -0.06 | 0.44 |  |
|  | (0.20) | (0.25) | (0.40) | (0.58) | (0.68) | (1.28) | (0.16) | (0.19) | (0.32) |  |
| Constant | 15.84*** | 15.95*** | 16.77*** | 13.05*** | 12.07** | 19.84*** | 3.69*** | 2.87* | 5.93*** |  |
|  | (1.66) | (2.08) | (2.75) | (4.32) | (5.43) | (7.47) | (1.30) | (1.68) | (2.08) |  |
| Observations | 1076 | 798 | 278 | 1076 | 798 | 278 | 1076 | 798 | 278 |  |
| R^2^ | 0.23 | 0.21 | 0.28 | 0.36 | 0.30 | 0.48 | 0.35 | 0.32 | 0.41 |  |
